# BBB-PEP-prediction: improved computational model for identification of blood–brain barrier peptides using blending position relative composition specific features and ensemble modeling

**DOI:** 10.1186/s13321-023-00773-1

**Published:** 2023-11-18

**Authors:** Ansar Naseem, Fahad Alturise, Tamim Alkhalifah, Yaser Daanial Khan

**Affiliations:** 1 Department of Artificial Intelligence, School of Systems and Technology, University of Management and Technology, Lahore, Pakistan; 2 Department of Computer, College of Science and Arts in Ar Rass, Qassim University, Ar Rass, Saudi Arabia; 3 Department of Computer Science, School of Systems and Technology, University of Management and Technology, Lahore, Pakistan

**Keywords:** Machine Learning, Sequence Analysis, Ensemble Modeling, Peptide Classification, Transfer LearningArtificial intelligence, Data Mining, Supervised Learning, Pattern Recognition

## Abstract

BBPs have the potential to facilitate the delivery of drugs to the brain, opening up new avenues for the development of treatments targeting diseases of the central nervous system (CNS). The obstacle faced in central nervous system disorders stems from the formidable task of traversing the blood–brain barrier (BBB) for pharmaceutical agents. Nearly 98% of small molecule-based drugs and nearly 100% of large molecule-based drugs encounter difficulties in successfully penetrating the BBB. This importance leads to identification of these peptides, can help in healthcare systems. In this study, we proposed an improved intelligent computational model BBB-PEP-Prediction for identification of BBB peptides. Position and statistical moments based features have been computed for acquired benchmark dataset. Four types of ensembles such as bagging, boosting, stacking and blending have been utilized in the methodology section. Bagging employed Random Forest (RF) and Extra Trees (ET), Boosting utilizes XGBoost (XGB) and Light Gradient Boosting Machine (LGBM). Stacking uses ET and XGB as base learners, blending exploited LGBM and RF as base learners, while Logistic Regression (LR) has been applied as Meta learner for stacking and blending. Three classifiers such as LGBM, XGB and ET have been optimized by using Randomized search CV. Four types of testing such as self-consistency, independent set, cross-validation with 5 and 10 folds and jackknife test have been employed. Evaluation metrics such as Accuracy (ACC), Specificity (SPE), Sensitivity (SEN), Mathew’s correlation coefficient (MCC) have been utilized. The stacking of classifiers has shown best results in almost each testing. The stacking results for independent set testing exhibits accuracy, specificity, sensitivity and MCC score of 0.824, 0.911, 0.831 and 0.663 respectively. The proposed model BBB-PEP-Prediction shown superlative performance as compared to previous benchmark studies. The proposed system helps in future research and research community for in-silico identification of BBB peptides.

## Introduction

The BBB serves as a barrier that prevents infections, blood cells, and components of neurotoxic plasma from entering the brain [1]. Blood vessels play a crucial role in supplying oxygen and essential nutrients to all tissues and organs in the body [2]. When it comes to the CNS, the blood vessels that vascularized it possess distinct characteristics known as the blood–brain barrier [3]. This barrier enables the tight regulation of ion, molecule, and cell movement between the bloodstream and the brain. By maintaining precise control over CNS homeostasis, the blood–brain barrier ensures optimal neuronal function and safeguards neural tissue from harmful toxins and pathogens. Any changes to the integrity of this barrier are significant factors in the development and progression of various neurological disorders [4]. The presence of barrier layers at critical interfaces between blood and neural tissue plays a vital role in regulating the processes involved [2].

Blood–brain barrier penetrating peptides (BBPs) have the ability to traverse the blood–brain barrier through diverse mechanisms, without compromising its integrity [5]. Neurons are protected from hazardous compounds found in the bloodstream by BBB, which acts as a barrier. Additionally, it is critical for maintaining the CNS carefully balanced internal environment, which is necessary for the proper operation of synapses and neurons. When the BBB is damaged, harmful substances like viruses, cells, and neurotoxic particles from the bloodstream can enter the brain. This may result in inflammatory and immunological responses, activating a number of pathways that support neurodegeneration [6].

Studies have shown that certain BBPs can facilitate the delivery of drugs into the brain, opening up new possibilities for the development of treatments targeting CNS disease [7]. The impasse observed in CNS disorders arises from the significant challenge of crossing the BBB for pharmaceutical agents. Approximately 98% of small molecule-based drugs and nearly 100% of large molecule-based drugs are unable to penetrate the BBB successfully [8].

In the proposed study, the contribution have been made are listed below.

1. The collected benchmark dataset has been fed to novel feature computation approaches such PRIM, RPRIM, AAPIV, RAAPIV and FV.
2. Statistical moments such as Raw, Hahn and central have been employed.
3. Four types of ensembles such as bagging, boosting, stacking and blending have been utilized for modeling purposes.
4. Bagging employed RF and ET, while Boosting utilizes XGB and LGBM. Stacking uses ET and XGB as base learners, blending exploited LGBM and RF as base learners, while LR has been applied as Meta learner for stacking and blending.
5. Four types of tests such as self-consistency, independent set, cross validation with 5 and tenfold and jackknife test have been accomplished.
6. Evaluation metrics such as Accuracy, specificity, sensitivity and MCC have been used for evaluation of proposed model.

For the computational identification of blood–brain barrier peptides, just a few studies have been conducted. Dai et al. has conducted a study on predicting BBB peptides, where feature selection has been utilized by discarding redundant and irrelevant features. Finally, logistic regression has employed prediction of BBB peptides [9]. Another study contributed by extending the dataset and usage of several feature descriptors. The researcher has used several machine-learning approaches such as Decision Tree, Random forest, Logistic Regression, KNN and Gaussian Naive Bayes (GNB), XGB, and Support Vector Classifier (SVC) for identification of BBB peptides [10].

The latest benchmark study by Chen et al. has been incorporated by extending the data. The study uses CKSAAP and PAAC as feature vectors and DT, RF, KNN, AdaBoost, GentleBoost, LogitBoost, linearSVM and rbfSVM to predict blood–brain barrier peptides [11].

## Materials and methods

This section explores the dataset used to conduct study, and employs classifiers to predict BBB penetrating peptides. The first section describes the data acquisition; the second section explores the feature generation process. Finally, the last elaborates the employed classifiers approaches.

**Fig. 1:**
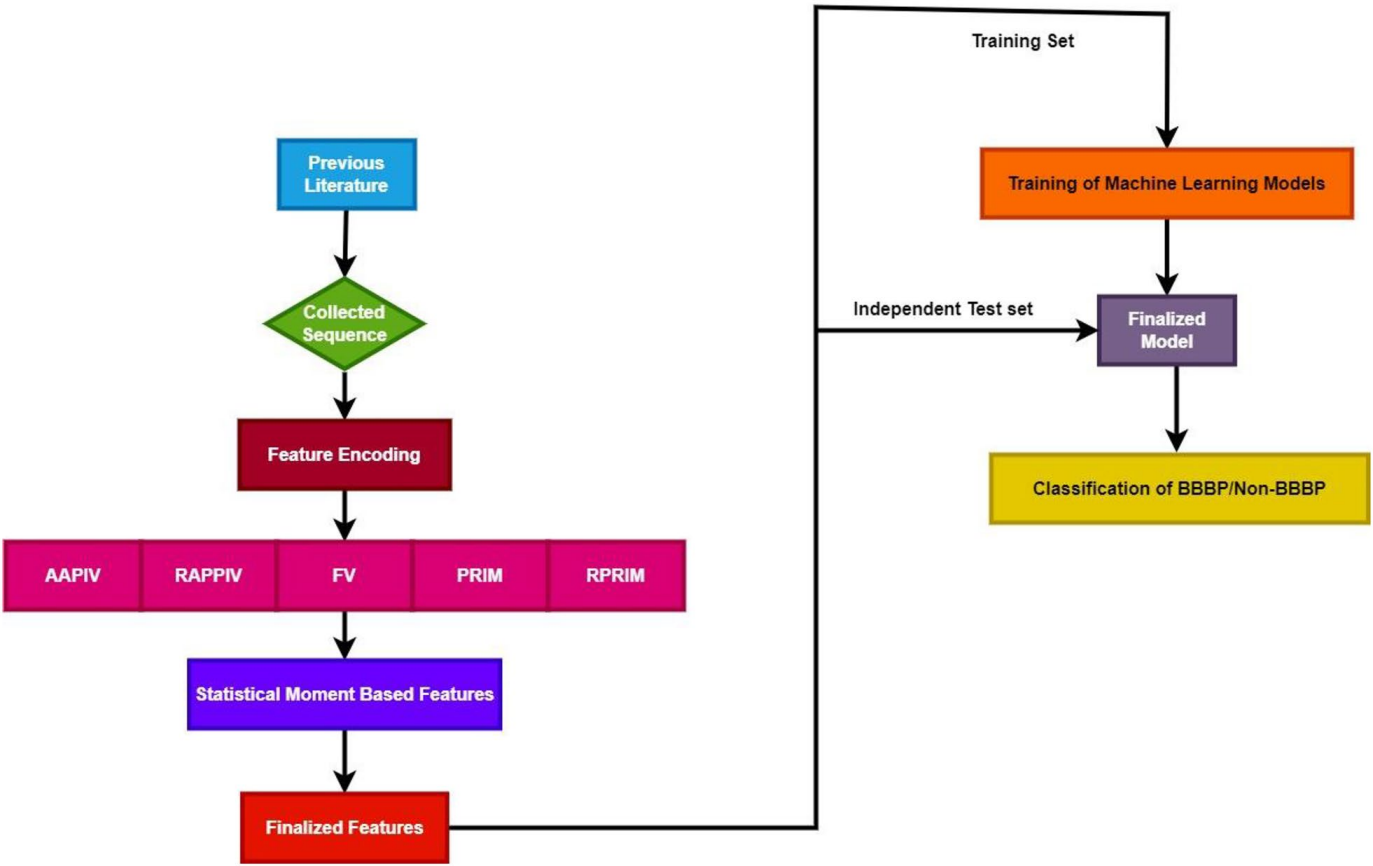
Architecture employed in this study

Figure 1 shows the architecture employed for identification of Blood–brain barrier penetrating peptides. The position based and statistical moments based features have been computed and fed to machine learning classifiers for training and test purpose.

### Dataset description

The benchmark dataset has been collected from Chen et al. [11]. The experimentally validated Blood–Brain Barrier Peptides (BBPs dataset has been acquired from different research papers such as Dorpe et al. [12], B3Pdb Kumar et al. [13], public datasets of BBPpred Dai et al. [9], B3Pred Kumar et al. [10]. For the collection of non-BBPs, sequences were obtained from UniProt using specific query criteria to exclude peptides related to blood–brain barrier, brain, Brainpeps, B3Pdb, permeation, permeability, venom, toxin, transmembrane, transport, transfer, membrane, neuro, and hemolysis. Redundant sequences were then removed using CD-HIT with a sequence identity cut-off of 10% Dai et al. [9]. Finally, peptide sequences with ambiguous residues were also excluded. This process yielded 425 non-BBPs. Overall dataset consists of 425 positive samples and 425 negative samples. The feature vectors have been generated using the combined dataset based on positive and negative sequences. The hyper-parameters tuning has been performed for three classifiers on the entire dataset to exhibit better results. Once the optimal hyper-parameters have been originated, the dataset has been split into 77 and 23% for training and test set respectively.

### Feature formulation

Position variant and composition-specific feature extraction techniques are employed to extract features from proteomic and genomic sequences. These widely recognized techniques consist of the following components.

#### Position relative incidence matrix (PRIM)

The arrangement of amino acid residues within the polypeptide chain holds significant importance in unraveling the hidden properties of the protein. To unveil intricate patterns formed by the placement of residues, a matrix is created to capture positional correlations among all residues [14]. This matrix, known as PRIM (Positional Residue Interaction Matrix), is designed as a 20 × 20 grid to estimate the positional information of the protein [15], considering the twenty unique amino acid residues present in each polypeptide chain [16].

1 $${R}_{PRIM}=\left[\begin{array}{cccccc}{R}_{1\to 1}& {R}_{1\to 2}& \cdots & {R}_{1\to y}& \cdots & {R}_{1\to 20}\\ {R}_{2\to 1 }& {R}_{2\to 2}& \cdots & {R}_{2\to y}& \cdots & {R}_{2\to 20}\\ \vdots & \vdots & & \vdots & & \vdots \\ {R}_{x\to 1}& {R}_{x\to 2}& \cdots & {R}_{x\to y}& \cdots & {R}_{i\to 20}\\ \vdots & \vdots & & \vdots & & \vdots \\ {R}_{A\to 1}& {R}_{A\to 2}& \cdots & {R}_{A\to y}& \cdots & {R}_{A\to 20}\end{array}\right]$$

Each element (Rij) within the PRIM matrix represents the sum calculated based on the relative position of the ith residue with respect to the jth residue, indicating the presence of the ith residue at that position. Consequently, the resulting matrix comprises 400 coefficients. To mitigate the complexity of dimensions, statistical moments are calculated, resulting in a set of 30 enumerated features derived from the original 400-coefficient matrix [17].

#### Reverse position relative incidence matrix (RPRIM)

The Reverse Position Relative Incidence Matrix (RPRIM) is an enumeration technique that shares similarities with the aforementioned method, but it delves deeper into uncovering hidden features of sequences that exhibit homologous peculiarities. RPRIM is calculated by utilizing the reverse sequence of the original sequence [18]. The resulting RPRIM matrix, computed through this process, is provided below.

2 $${R}_{RPRIM}=\left[\begin{array}{cccccc}{Q}_{1\to 1}& {Q}_{1\to 2}& \cdots & {Q}_{1\to y}& \cdots & {Q}_{1\to 20}\\ {Q}_{2\to 1 }& {Q}_{2\to 2}& \cdots & {Q}_{2\to y}& \cdots & {Q}_{2\to 20}\\ \vdots & \vdots & & \vdots & & \vdots \\ {Q}_{x\to 1}& {Q}_{x\to 2}& \cdots & {Q}_{x\to y}& \cdots & {Q}_{i\to 20}\\ \vdots & \vdots & & \vdots & & \vdots \\ {Q}_{A\to 1}& {Q}_{A\to 2}& \cdots & {Q}_{A\to y}& \cdots & {Q}_{A\to 20}\end{array}\right]$$

Similar to PRIM, the RPRIM matrix also consists of 400 coefficients, maintaining the same dimensionality. However, through the application of statistical moments, the dimensionality of RPRIM is subsequently reduced to 30 coefficients, just like in the case of PRIM [15].

#### Frequency vector (FV)

The frequency vector is a valuable source of information that reveals the distribution of residues within a polypeptide chain in a given sequence [19]. It calculates the occurrence rate of individual residues in the protein. The FV characteristic ensures that details about the composition and distribution of protein sequences are retained. The FV is represented as follows.

3 $$FV \, = \, \left[ {f_{1} ,f_{2} ,f_{3} , \ldots ,f_{20} } \right]$$

The FV is a vector with 20 dimensions that calculates the frequency of each amino acid residue in the sequence, based on their alphabetic ordinal value.

#### Accumulative absolute position incidence vector (AAPIV)

The FV captures the distributional details of each amino acid residue in a protein and identifies ambiguous features related to its composition. However, the FV does not include information about the relative positions of the amino acid residues. To address this, the AAPIV (Amino Acid Positional Information Vector) was introduced, which partitions the relative positional information into four quarters [20]. This information is computed based on the occurrence of the 20 native amino acids, as shown below.

4 $$K \, = \, \left[ {\forall_{1} ,\forall_{2} ,\forall_{3} , \ldots ,\forall_{n} } \right]$$

where the *i*^*th*^ section of AAPIV is calculated as

5 $${\forall }_{i}={{\Sigma }^{n}}_{k=1} {\beta }_{k}$$

Considering a specific nucleotide, k represents a randomly chosen location. In the AAPIV, a designated component, denoted as I, accumulates the sum of all the locations where the ith nucleotide occurs.

#### Reverse accumulative absolute position incidence vector (RAAPIV)

RAAPIV shares similarities with AAPIV, with the key distinction being that it utilizes the reverse sequence of the original sample to generate the output vector. This reversal enables the extraction of additional knowledge regarding positional information, allowing for the discovery of concealed and profound characteristics within the sequences [21]. The vector is represented as follows.

6 $$RAAPIV = \left[ {n_{1} ,n_{2} ,n_{3} , \ldots ,n_{m} } \right]$$

### Statistical moments

The feature set is populated with the raw, Hahn, and central moments of the genomic data, which contribute essential elements to the input vector for the model. Researchers have recognized that the characteristics of proteomic and genomic sequences depend on both the composition and the relative positions of their bases. Consequently, computational and mathematical models have focused on capturing the correlated placement of nucleotide bases in genomic sequences to enhance the feature vector [22]. This attention to correlated positioning is crucial for establishing a reliable and comprehensive feature set [23].

Hahn moments require two-dimensional data, so genomic sequences are transformed into a two-dimensional matrix S’ with dimensions k*k, which contains the same information as matrix S but arranged in a two-dimensional format. Therefore,

7 $${\text{k }} = \, \surd {\text{n}}$$

8 $$S{\prime} = \left| {S_{11} S_{12} \ldots S_{1n} S_{21 . . . } S_{22 . . . } \ldots S_{2n . . . } S_{n1} S_{n2} \ldots S_{nn} } \right|$$

In order to reduce dimensionality, statistical moments are computed based on the obtained square matrix, resulting in the creation of a fixed-size feature vector [28]. As previously mentioned, this study employs Hahn, central, and raw moments for this purpose.

The below equation entitles the calculation of raw moments of order a + b.

9 $${U}_{ab}={{\Sigma }^{n}}_{e=1}{{\Sigma }^{n}}_{f=1} {e}^{a}{f}^{b}\delta ef$$

The sequences contain significant information embedded within their Moments, specifically up to the third order are ${U}_{00}{, U}_{10}$*,*${U}_{11}$*,*${U}_{20}$*, *${U}_{02}$*, *${U}_{21}$*,* ${U}_{12, }{U}_{03}$ and ${U}_{30}$. To compute the central moments ($\underline{x}\underline{y}$), it is necessary to calculate the centroid first, which represents the central point of the data [24]. The central moments are then computed using this centroid according to the following procedure:

10 $${v}_{ab}={{\Sigma }^{n}}_{e=1}{{\Sigma }^{n}}_{f=1) }{\left(e-\underline{x}\right)}^{a}{\left(f-\underline{y}\right)}^{b}\delta ef$$

The computation of Hahn moments involves the use of a square grid as the discrete input. This choice helps elucidate both the regularity and reversibility of the data, as the original data can be reconstructed using inverse Hahn moments. Due to the reversibility property of Hahn moments, the information transformed from the original sequences remains intact and is incorporated into the model through the feature vector [15]. The computation of Hahn moments is depicted by the equation provided below.

11 $${h}_{n}^{x,y}\left(p,Q\right)=(Q+{V-1)}_{n}(Q{-1)}_{n}\times {{\Sigma }^{n}}_{z=0}{\left(-1\right)}^{z}\frac{({-n)}_{z}({-p)}_{z}({2Q+x+y-n-1)}_{z}}{({Q+y-1)}_{z}(Q{-1)}_{z}}\frac{1}{z!}$$

The equation utilizes Pochhammer notation and the Gamma operator, which are explained in detail by Akmal et al. [25].

The Hahn coefficients obtained from the previous equation are typically normalized using the coefficients specified in the subsequent equation.

12 $${H}_{pq}= {{\Sigma }^{G-1}}_{j=0}{{\Sigma }^{G-1}}_{i=0} {\delta }_{pq}{{h}^{a,b}}_{p}\left(j,Q\right) {{h}^{a,b}}_{q} \left(i,Q\right), m,n=\mathrm{0,1},2, \dots , Q-1$$

### Classification models

This section offers an overview of the classification algorithms utilized in this study. Various ensemble methods, including bagging, boosting, blending, and stacking, were employed. Additionally, the algorithms underwent evaluation and comparative analysis to assess their performance.

#### Bagging

**Fig. 2:**
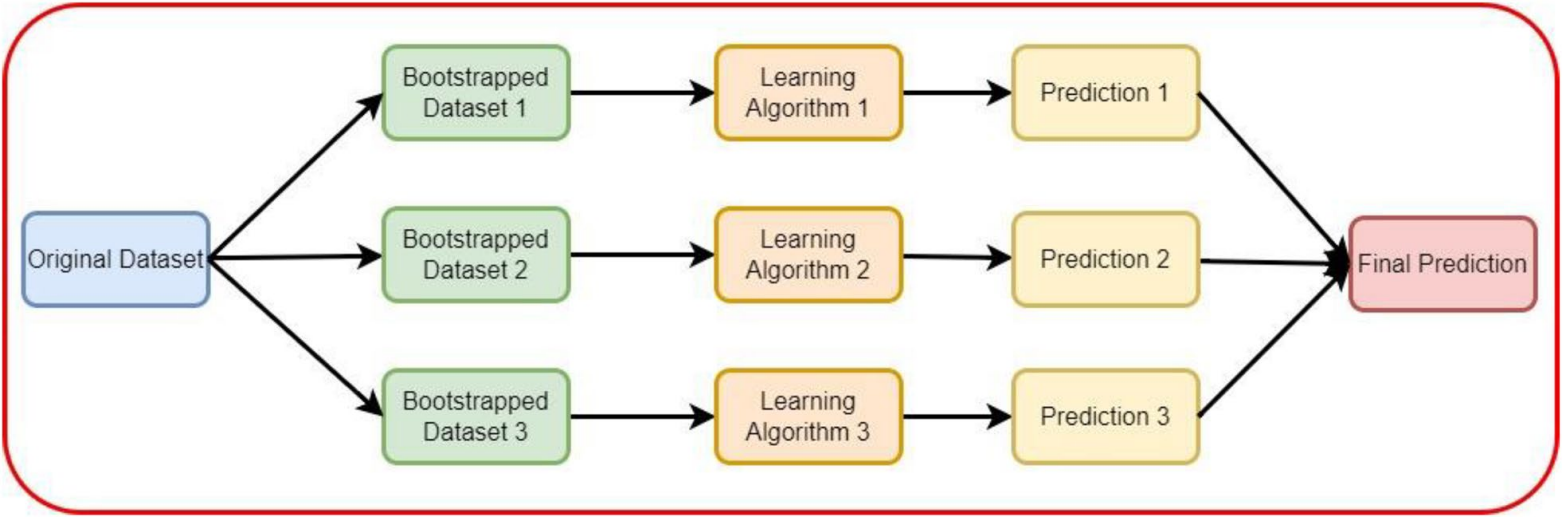
Bagging architecture used in this study

Bagging, an ensemble-based approach, is commonly employed for diverse machine learning problems. It operates in a parallel manner, dividing the dataset into multiple subsets using sampling with replacement [26]. In this study, two classifiers were utilized within the bagging approach. Figure 2 illustrates the architecture of the bagging methodology.

Figure 2 illustrates the structural framework of the bagging methodology utilized for predicting BBB peptides.

##### Extra trees

ET, belonging to the bagging family of algorithms, shares similarities with the random forest algorithm but has two key differences. During training, the ET model receives positive and negative sequences, along with their respective labels. By dividing the tweets into subsets of equal size and creating various sub-datasets based on positive and negative sequences, multiple decision trees are generated [27]. Each decision tree is built with a fixed number of split nodes. For a given test tweet, each weak learner model predicts its class, and the class prediction with the highest number of votes determines the classification for the test sample. For accuracy enhancement, the hyper-parameters optimization for ET has been employed.

Table 1 shows the hyper-parameters values after optimization. Randomized search CV has been used to find optimal parameters.

**Table 1:** Hyper-parameter optimization for ET

Classifier	n_estimators	min_samples_split	max_depth
ET	100	2	None

##### Random forest

RF has been utilized in various computational proteomics and genetics problems [28]. RF utilizes a bootstrapping strategy for sample distribution and is a member of the bagging family. The model initially builds sub-datasets of positive and negative samples using sampling with replacements [29]. The sequences are distributed equally throughout each subset. A feature vector with a label for training purposes and decision trees made with randomly selected best-split nodes serve as the model’s sources of information. All weak learners receive a test instance, and the class prediction is decided by a majority vote.

#### Boosting

Another ensemble strategy that uses an iterative learning process is boosting. Unlike bagging, the dataset is not broken up into smaller sub-datasets; rather, all of the learners work in a serial fashion. Each classifier in boosting algorithms educated on data while accounting for the output of earlier weak learners. After each epoch, the weights are dispersed once more. In the succeeding learners, only incorrectly classified observations are considered, and the incorrectly classified samples are given a high weight up to a specified number of epochs. The classification of all test samples as positive or negative is accomplished by repeating this process [30]. The description of each boosting algorithm is explained in the next section.

**Fig. 3:**
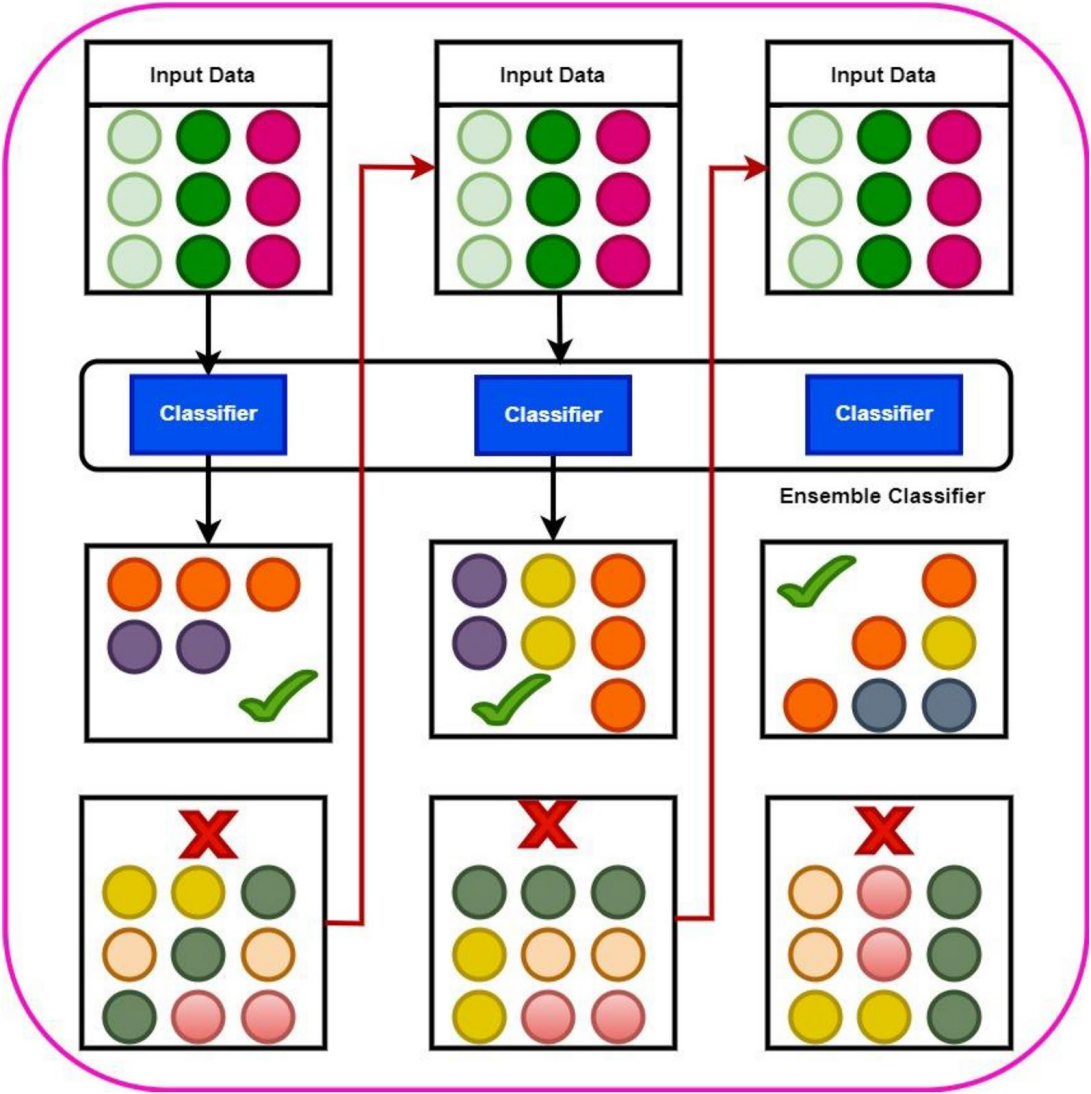
Boosting architecture

Figure 3 illustrates the structure of the boosting ensemble used for the identification of BBB peptides.

##### XGBoost

The XGB boosting algorithm builds decision trees that are divided into sections along the tree’s depth, and it is trained and evaluated on a dataset using these decision trees. The number of trees in algorithms is specified by the default number of weak learners. The first tree was made using the provided data, and test data was used to evaluate the model. The incorrectly identified observations were given a lot of weight and were passed on to the weaker learner after them. In this case, the tree is exclusively formed using incorrectly identified samples [31]. The model completes its performance in this manner.

##### LGBM

Following model execution, the light gradient boosting machine splits the decision tree leaf-wise. The leaf that performs best and has the lowest error is chosen by LGBM. A large weight is given to occurrences that were mistakenly identified in the sequential process, and the output from the first weak learner is transferred to the second weak learner [32]. Until the model produces the best results or the maximum number of iterations have been accomplished, this process is repeated.

The tuned hyper-parameters values for boosting classifiers, obtained by Randomized Search CV, are presented in Table 2.

**Table 2:** Hyperperameters tuning for boosting classifiers

Classifier	n_estimators	max_depth	learning_rate
XGB	400	9	0.1
LGBM	200	5	0.1

#### Stacking

The stacking approach, which uses multiple heterogeneous classifiers instead of bagging and boosting, also makes use of the two layers notion [33]. In the first layer, base learners ET and XGB have been used. On the entire dataset using cross validation, both classifiers have been trained and produced the predations. A newly constructed 2-D dataset is further divided into training and test set. The Meta classifier LR has been trained by using training data based on prediction from level 0 and tested on test data.

**Fig. 4:**
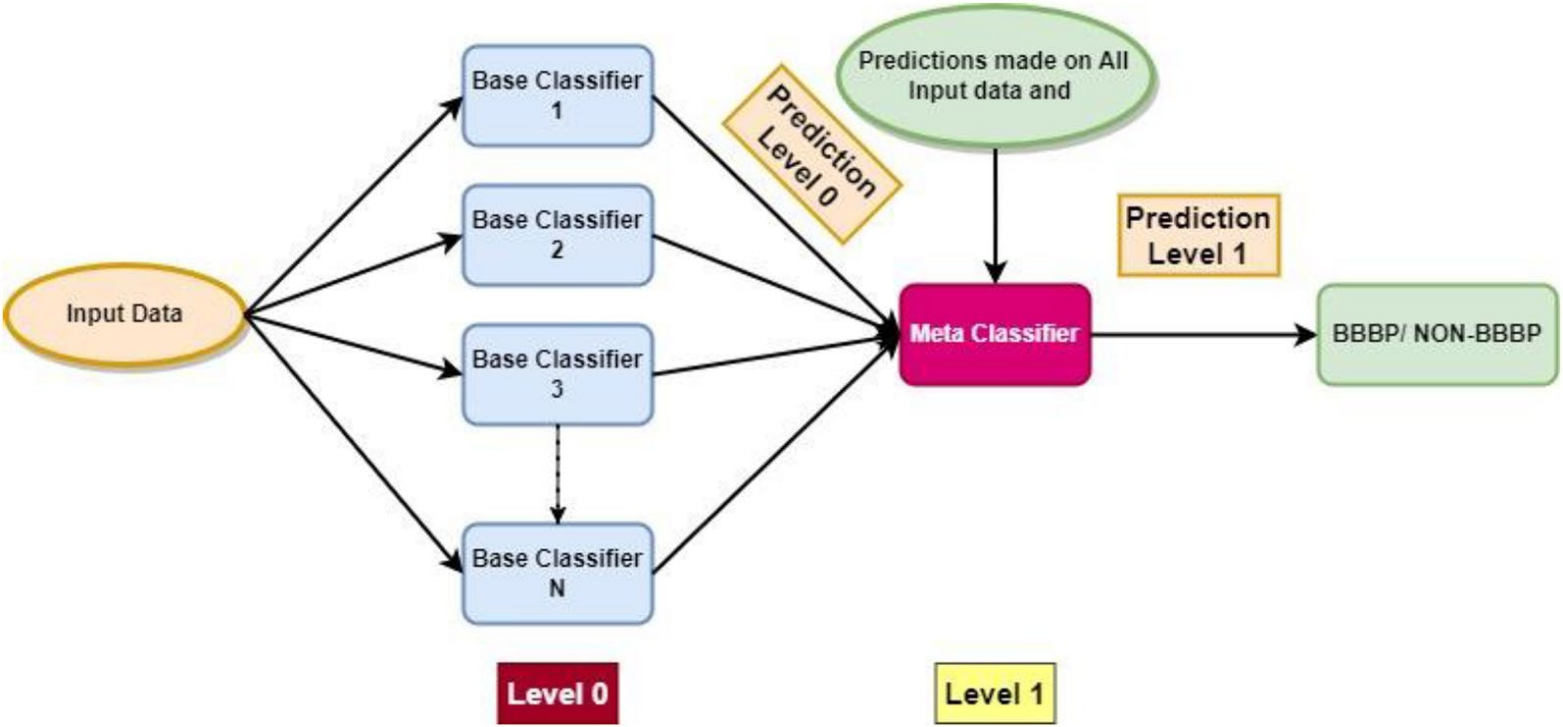
Stacking architecture

Figure 4 demonstrates the architecture of the stacking ensemble employed for the prediction of BBB peptides.

##### Logistic regression

Contemporary machine learning algorithm for supervised learning called LR has been used to solve many in-silico proteomics difficulties. To get a projected score against each sample, the weights and inputs are multiplied. Each anticipated score has been subjected to the sigmoid function, which produces values between 0 and 1 [29]. A decision border is constructed to separate two classes as a result. When a test sample appears to be classified, it will be placed in a positive class if the value obtained after applying the sigmoid is greater than 0.5 and a negative class if it is less than 0.5. The threshold is set at 0.5.

13 $$P=\frac{1}{1+{e}^{-z}}$$

where z is

14 $$\mathrm{z }=\left({w}_{1}{x}_{1}+{w}_{2}{x}_{2}+\dots {w}_{n}{x}_{n}+b\right)$$

The linear portion of the sigmoid activation function is elaborated in Eq. 14, where w’s represents the initialized weights and x’s stands for the inputs from the data.

#### Blending

Blending is an ensemble approach, which combines several heterogeneous classifiers [34]. The validation dataset including train and test data, as well as the blending approach, are in opposition to stacking. With validation data that has been extracted from the training set in the first layer, the train and test data are divided. Predictions were produced on the test set and validation set using the used models RF and LGBM, which were trained on the training dataset. The prediction obtained from the validation set combined with the initial validation set creates a new dataset in the second layer. The recently created dataset was tested on test data divided into the first layer and trained using the Meta classifier LR.

**Fig. 5:**
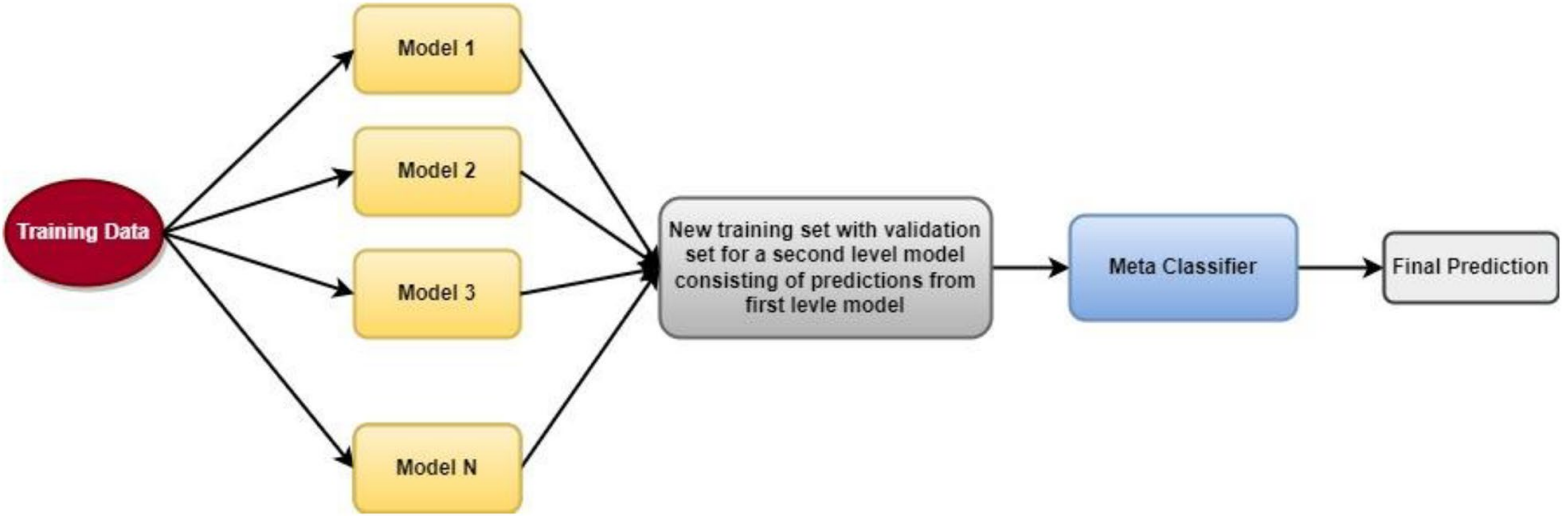
Blending architecture

The structure of the blending ensemble utilized for the identification of BBB peptides is demonstrated in Fig. 5.

### Evaluation metrics

The suggested model is evaluated using a variety of measures, including ACC score, SPE, SEN, and MCC. The ACC score shows the total number of samples from both classes that were correctly predicted out of all samples [35]. In order to quantify the negative cases that can be expected from the accuracy of the model, SPE has been used [36]. SEN shows how well the model can locate occurrences of positivity [37]. Despite unbalanced data, MCC is a reliable metric since it considers both classes [38]. If the model successfully detected both the positive and negative samples, it will produce an assiduous MCC score. For every discussed metric, the formulas are given.

15 $$Accuracy=\frac{TP+TN}{TP+TN+FP+FN}$$

16 $$Specificity=\frac{TN}{TN+FP}$$

17 $$Senstivity=\frac{TP}{TP+FN}$$

18 $$MCC=\frac{TN\times TP-FN \times FP}{\sqrt{(FP+TP)(FN+TP)(FP+TN)(FN+TN)}}$$

A True Positive represents the Peptides belonging to the positive class that has been accurately identified by the predictor. False Negative refers to the Peptides that belongs to positive class but are negatively identified by the predictor. False Positive, on the other hand, it indicates Negative samples but are identified as Positive samples by the predictor. A True Negative corresponds to the samples belonging to the negative class that is correctly identified by the predictor.

## Results and discussion

Four types of rigorous tests, including the self-consistency test, independent set test, K-fold with 5 and tenfold cross-validation, and jackknife testing, were carried out to evaluate predictor robustness.

### Self-consistency

A simple test that is frequently used to assess a predictor’s accuracy is the self-consistency test. Without performing a train-test split, the model was trained on the entire set of data to ensure that the prediction was self-consistent. Following training, the model is tested against the training dataset to determine whether all classifiers have formed the model appropriately [39]. Since the model was trained and tested using the same dataset, the self-consistency test illustrates the consistency of the model with respect to its data [40]. Table 1 displays the results of self-consistency.

Table 3 presents the various techniques employed to test self-consistency, each of which has been demonstrated. All the employed methods exhibit perfect accuracy across all evaluation metrics, indicating that the predictor aligns consistently with the data.

**Table 3:** Self consistency results

Model	ACC	SEN	SPE	MCC
RF	0.998	0.998	0.998	0.966
ET	0.998	0.999	1	0.996
LGBM	0.998	0.998	0.998	0.996
XGB	0.998	0.998	0.998	0.996
Stacking (ET, XGB, LR)	0.998	0.998	0.996	0.995
Blended (LGBM, RF, LR)	0.998	0.998	0.98	0.995

**Fig. 6:**
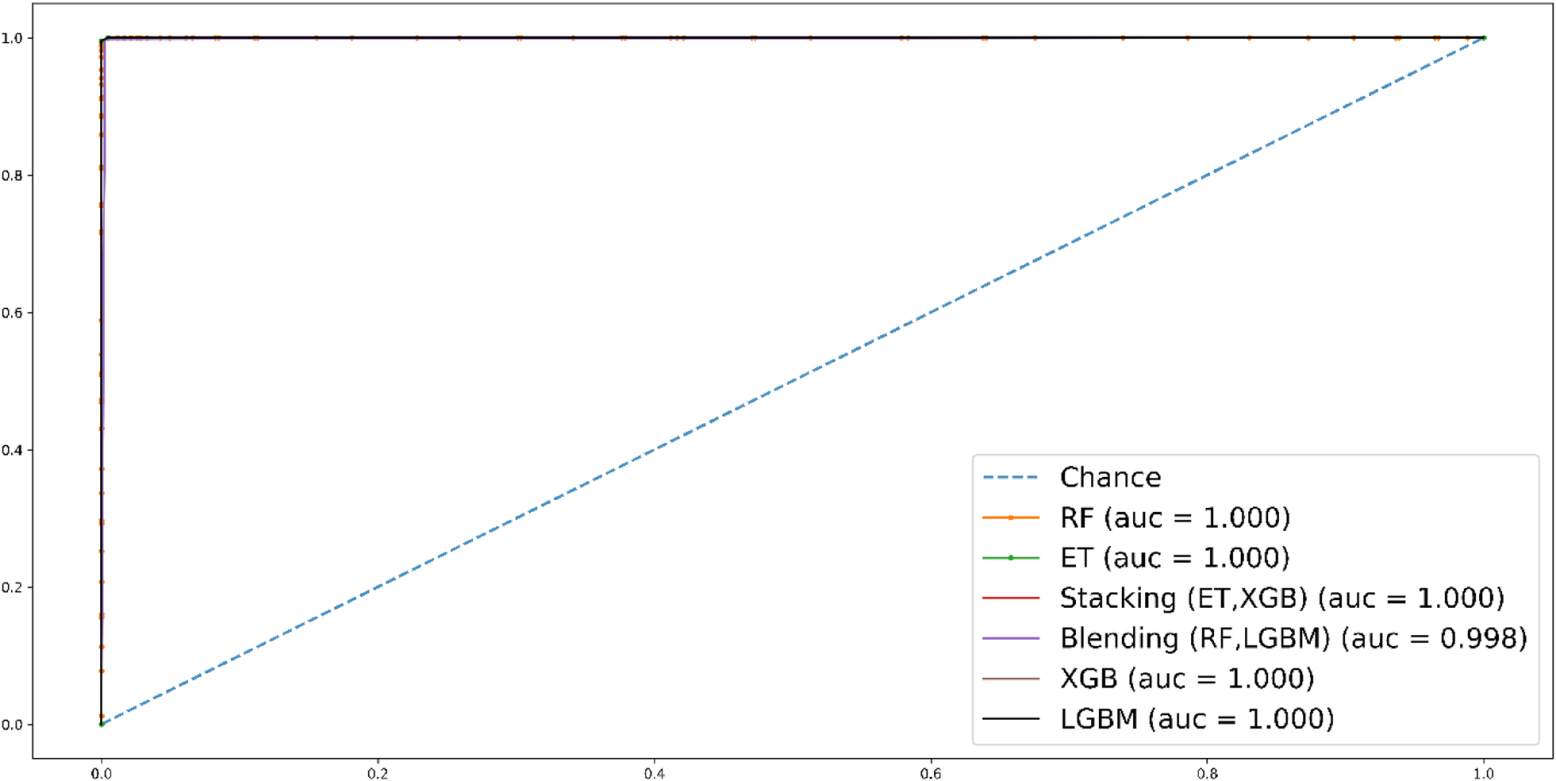
Roc curve for self-consistency test

Following that, a ROC curve was utilized to evaluate the accuracy of each predictor. The findings reveal that all the predictors achieved a perfect score of 100% except Blending of classifiers. Figure 6 visually represents this, showcasing that all the predictors attained the highest area under the curve score.

### Independent testing

Another method for evaluating the performance of a predictor with unseen data is through independent testing. Typically, the data is divided into two parts for this evaluation [22]. The first part, constituting 77% of the entire dataset, is allocated to the training set. In this set, input and output pairs are provided to the model to facilitate accurate learning. The remaining 23% is used to assess the predictor's performance. In this testing phase, only input features are provided to the predictor, while the class label remains unknown. The predictor makes predictions on this unseen data, which was not exposed during the training phase [41]. All the evaluation measures discussed are presented in Table 2 for the employed classifiers.

According to the data presented in Table 4, the Stacking classifier demonstrates remarkable performance with MCC and accuracy scores of 0.663 and 0.824, respectively. The results obtained from testing on an independent dataset signify that the predictor performs well on unseen data, which was not encountered by the predictor during the training phase.

**Table 4:** Results from the independent set

Model	ACC	SEN	SPE	MCC
RF	0.769	0.775	0.844	0.550
ET	0.789	0.798	0.9	0.600
LGBM	0.789	0.796	0.867	0.590
XGB	0.809	0.816	0.9	0.634
Stacking (ET, XGB, LR)	0.824	0.831	0.911	0.663
Blended (LGBM, RF, LR)	0.804	0.806	0.822	0.609

**Fig. 7:**
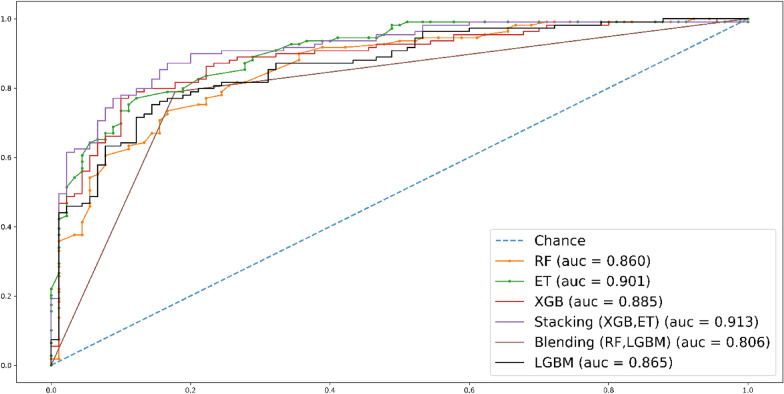
Roc curve for independent set test

During independent-set testing, it is evident that stacking classifier surpass other predictors, showcasing superior performance compared to all other methods. The results are visually depicted in Fig. 7.

### Cross validation

Cross-validation is a distinct testing technique that operates differently compared to self-consistency and independent testing. In self-consistency testing, the predictor doesn’t make predictions on unseen data since all the available data is utilized for training and the same-trained data is used for testing [42]. This limitation necessitates the use of independent testing to assess the performance of the predictor on unseen data. However, when independent set testing is conducted on randomly distributed data, there is a chance that a considerable portion of the data may be overlooked [43]. To address this issue, cross-validation has been developed as a novel testing approach.

#### Fivefold cross-validation

Cross-validation is an extensive testing method that is applied to all samples [44]. It involves dividing the data into k-folds, where the value of k can vary but is typically set to 5 or 10 in the literature. For example, when k = 5, the data is partitioned into 5 equal parts. In each iteration, one fold is left out for testing, while the remaining four folds are used for training. This process is repeated until each fold has been used as a test set. The accuracy of each fold is computed, and the average accuracy is calculated as the final result. This approach ensures that all the data is both trained and tested in a disjoint manner. The outcomes for each classifier are presented in Table 3.

Among the employed methods, the stacking approach has demonstrated superior performance in the fivefold cross-validation (CV) testing. It has achieved impressive accuracy and MCC scores of 0.808 and 0.616, respectively. The results obtained from fivefold CV provide valuable insights into the effectiveness of the predictor as shown in Table 5. In contrast, independent set testing may have missed some data that could potentially be crucial for the predictor's learning. Thus, the fivefold CV approach has shown better results when compared to independent set testing.

**Table 5:** Results from 5 fold CV test

Model	ACC	SEN	SPE	MCC
RF	0.749	0.71	1	0.505
ET	0.808	0.752	1	0.620
LGBM	0.783	0.773	1	0.568
XGB	0.797	0.807	1	0.596
Stacking (ET, XGB, LR)	0.808	0.796	0.806	0.616
Blended (LGBM, RF, LR)	0.8	0.746	0.850	0.601

**Fig. 8:**
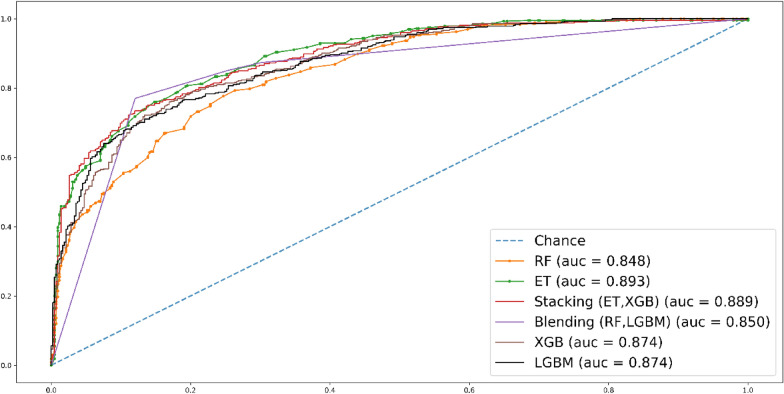
Roc curve for fivefold CV test

In Fig. 8, the fivefold cross-validation (CV) AUC results are presented. Stacking classifier stands out among the other methods, achieving a remarkable roc-AUC score of 0.889.

**Fig. 9:**
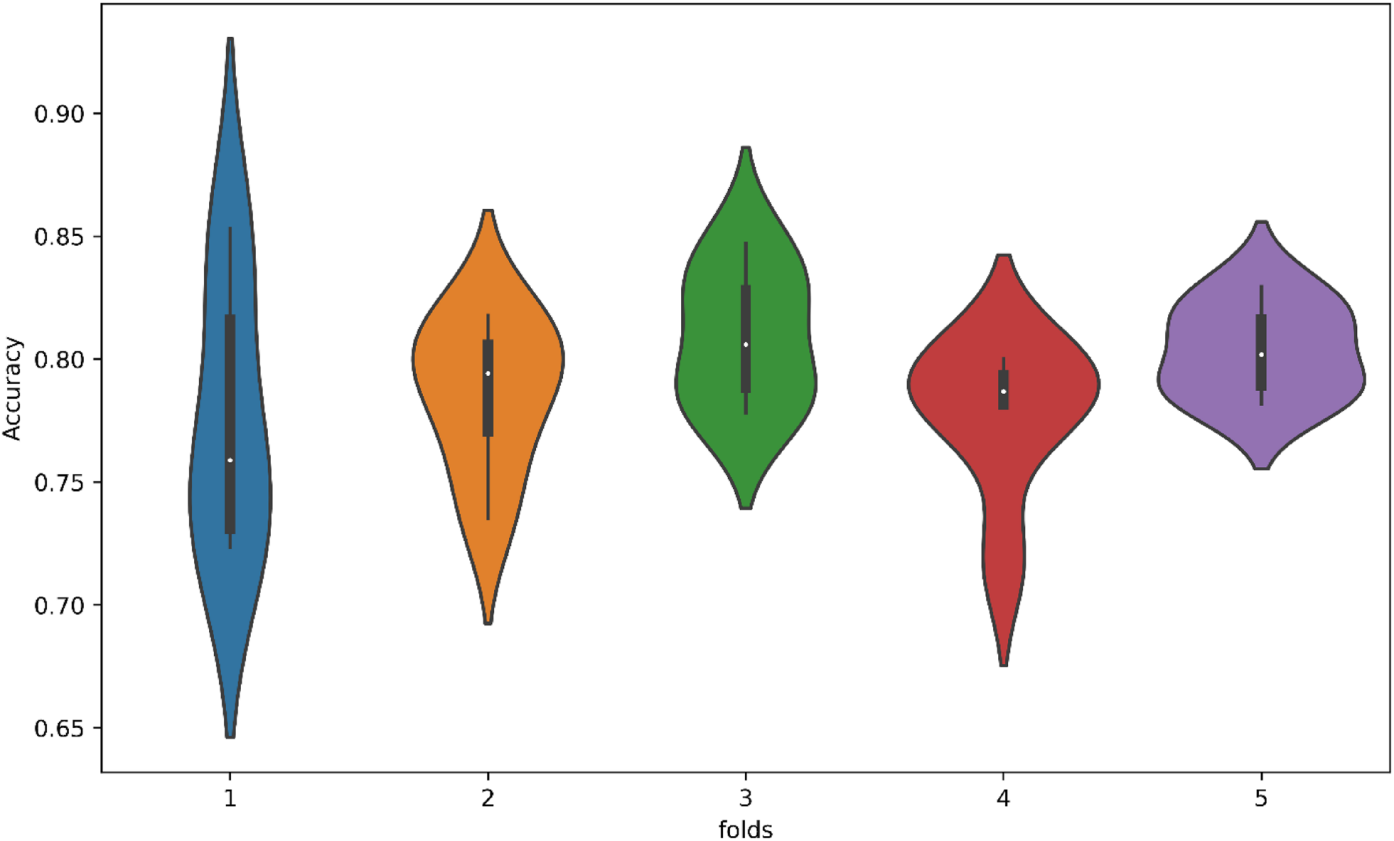
Violin chart for fivefold CV test

The violin plot combines the characteristics of boxplots and kernel density plots to visually represent the distributions of different groups. In Fig. 9, a violin chart displaying fivefold cross-validation results in terms of accuracy is presented. It is evident that each fold's accuracy can be attributed to each classifier. Notably, each classifier exhibits the highest accuracy in fold 1 and the lowest accuracy in fold 4.

#### Tenfold cross validation

In tenfold cross-validation, the dataset is divided into 10 equal-sized parts. This methodology involves training and testing ten models on these sub-datasets in a disjoint manner. In the first iteration, the first fold is used as the test set, while the remaining folds are utilized for training [19]. The accuracy score, specificity, sensitivity, and MCC score are calculated for this particular fold. Similarly, the models are tested on the second fold and trained on the remaining folds, and the evaluation metrics are computed. This process is repeated for each fold, ensuring that all folds are validated. Finally, the average of all the evaluation metrics is calculated to determine and report the overall performance.

The results obtained from the tenfold cross-validation (CV) test are presented in Table 6. The XGB classifier demonstrates superior performance compared to the other classifiers, achieving an accuracy score of 0.802 and an MCC score of 0.610. These results from the tenfold CV test surpass the results obtained from the fivefold CV test, indicating improved performance and consistency in the predictions.

**Table 6:** Results from 10 fold CV test

Model	ACC	SEN	SPE	MCC
RF	0.770	0.747	1	0.543
ET	0.791	0.759	1	0.588
LGBM	0.796	0.787	1	0.593
XGB	0.802	0.786	1	0.610
Stacking (ET, XGB, LR)	0.801	0.781	0.809	0.604
Blended (LGBM, RF, LR)	0.791	0.759	0.827	0.588

**Fig. 10:**
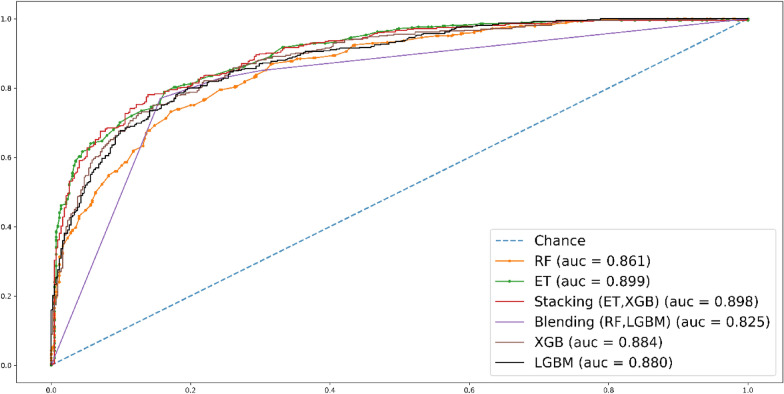
Roc curve for tenfold CV test

In Fig. 10, the AUC scores for each classifier in the tenfold cross-validation (CV) are displayed. ET stands out with a commendable AUC score of 0.889, closely followed by stacking with an AUC score of 0.898 and XGB with an AUC score of 0.884. These scores highlight the strong performance of these classifiers in terms of predictive accuracy and discrimination.

**Fig. 11:**
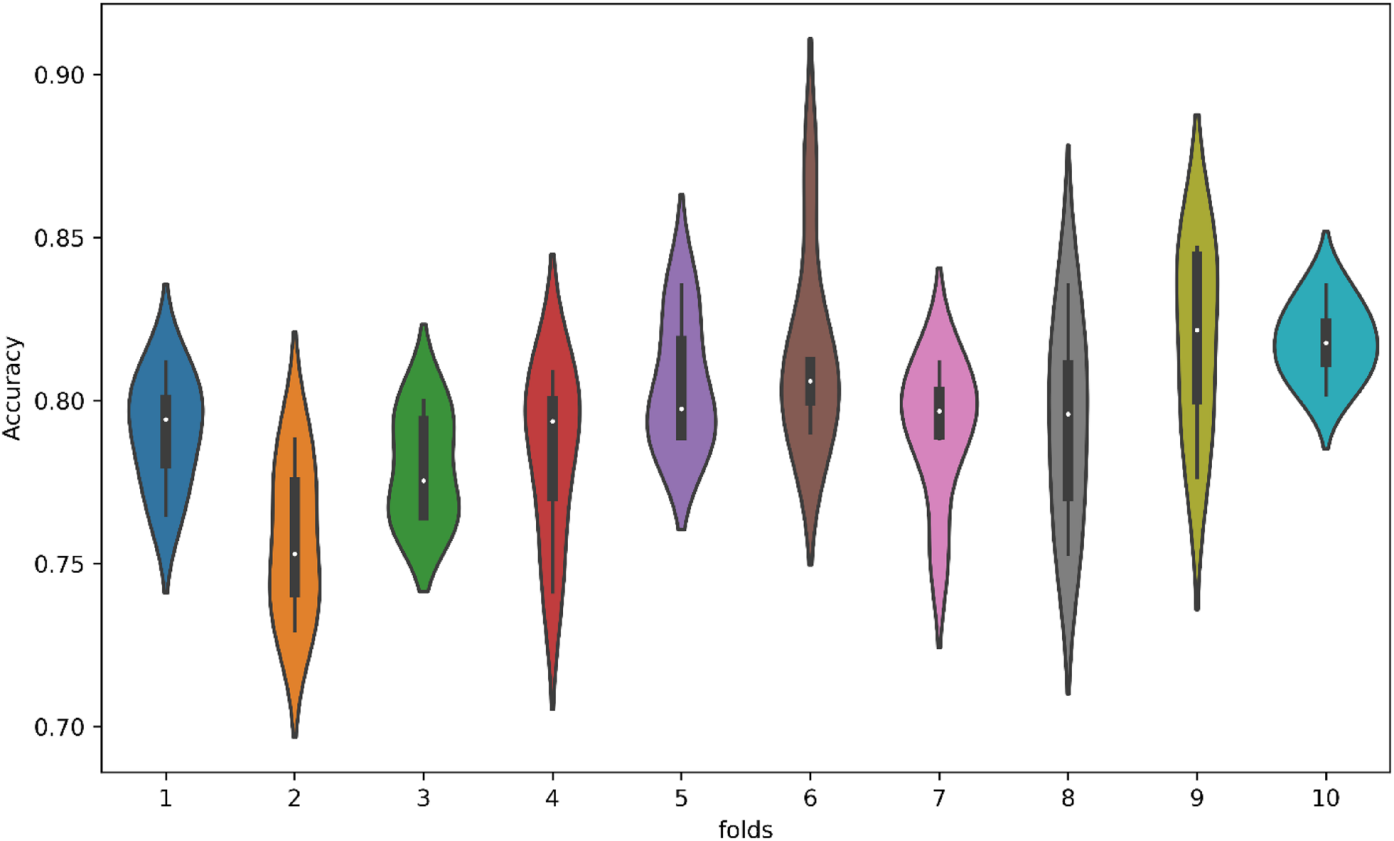
Violin chart for tenfold CV test

Figure 11 presents the results of the tenfold cross-validation, showcasing the excellent performance of the models. Fold 6 demonstrates the highest accuracy, while fold 2 exhibits the lowest accuracy. Notably, the mean accuracy reflects consistent and favorable results, as evidenced by the small gap between the highest and lowest values. Overall, the tenfold cross-validation demonstrates the models' strong performance across different folds.

### Jackknife

The Jackknife method is a robust testing measure that divides a dataset into n-folds, where n represents the total number of samples [21]. In each iteration of the Jackknife process, one sample is selected as the test instance, and the remaining samples are used as the training set. This process continues until each sample has been used for testing once, while the rest of the samples are utilized for training. The results obtained from the Jackknife self-consistency testing are presented in Table 5 below.

Table 7 displays the results obtained from the jackknife testing. Among the employed methods, the stacking classifier has demonstrated the best performance, achieving an accuracy score of 0.828 and an MCC score of 0.657 for the jackknife test. This testing approach has yielded the most favorable results compared to all other testing methods employed in the analysis.

**Table 7:** 5Results from jackknife test

Model	ACC	SEN	SPE	MCC
RF	0.776	0.748	0.804	0.552
ET	0.817	0.785	0.849	0.636
LGBM	0.816	0.809	0.823	0.633
XGB	0.8	0.802	0.797	0.600
Stacking (ET, XGB, LR)	0.828	0.808	0.832	0.657
Blended (LGBM, RF, LR)	0.8	0.795	0.804	0.599

**Fig. 12:**
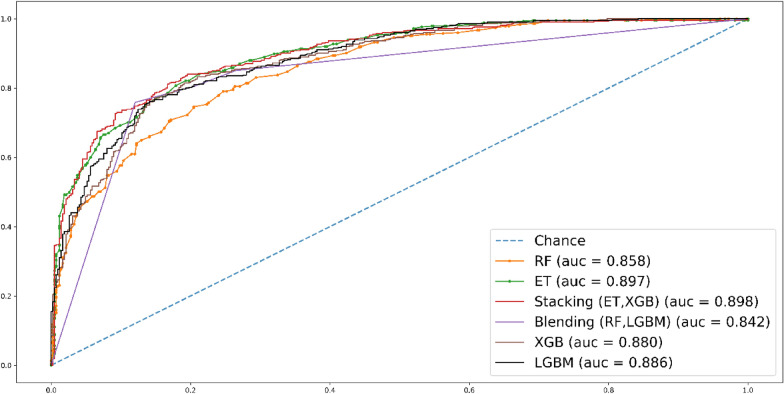
Roc curve for jackknife test

In Fig. 12, the ROC curve for the jackknife testing is presented. The ensemble-based bagging approach, stacking, outperforms the other methods, demonstrating superior performance in terms of the ROC curve.

Table 8 highlights that the proposed approach BBB-PEP-Prediction has achieved the best results when compared to existing state-of-the-art studies. These results have been obtained from independent set testing conducted in the proposed study.

**Table 8:** Comparison with previous studies

Author	Accuracy	Specificity	Sensitivity	MCC
BBPpred [9]	0.6667	0.6566	0.6768	0.3334
B3Pred [10]	0.6768	0.6465	0.7071	0.3542
BBBPredict [11]	0.7727	0.7778	0.7677	0.5455
BBB-PEP-prediction	0.824	0. 911	0.831	0.663

### Comparative analysis

This study utilized an in-silico method to identify interactions between BBB peptides. Human-constructed features were employed for peptides, utilizing position-specific and composition variant features to transform the sequences into enumerated forms. The resulting feature vectors were high-dimensional, so statistical moments such as Raw, Hahn, and central-based moments were used to reduce the dimensionality. This study extracted significant information about the properties of peptides, surpassing previous studies. State-of-the-art machine learning ensemble approaches including bagging, boosting, stacking, and blending were employed. RF and ET were used in the bagging approach, while XGB and LGBM were utilized in the boosting approach. Stacking involved ET and a XGB as base learners, with LR serving as the Meta learner. LGBM and RF were used as base learners, with LR as the Meta learner to reveal different patterns in order to identify the BBB peptides. LGBM, ET and XGB have been optimized by using Randomized Search CV to find the optimal parameters for accuracy enhancement. The employed classifiers effectively distinguished between both classes and the created feature space for BBB peptides demonstrated strong coefficients. Four types of testing were performed: self-consistency, independent set testing, cross-validation testing with 5 and 10 folds, and jackknife testing, to evaluate the predictor’s performance. Stacking classifiers consistently showed the best results across most of the tests, achieving an AUC score of 0.913, which outperformed existing methods. The accuracy scores for stacking in self-consistency, independent set testing, and cross-validation testing with 5 and 10 folds, as well as jackknife testing, were 1, 0.913, 0.889, 0.898, and 0.898, respectively. Compared with Chen et al. [11], who achieved the highest Accuracy score of 0.7727 in an independent set. Our proposed model BBB-PEP-Prediction achieved an improved Accuracy score of 0.824 for predicting BBB peptides. The computed features in this study were more robust and stringent in capturing the properties of sequences compared to other feature computation approaches. Overall, the results, particularly in cross-validation and jackknife tests, indicate a high level of generalization capability of the predictor. The complete results of the random forest experiment are shown below.

### Boundary visualization

In this section, we showcase the visualization of decision boundaries. When dealing with only two features, the decision boundary takes the form of a line that separates the samples belonging to one class from those of the other class. The visualization techniques employed in this study encompass boundary visualization for each classifier and raw sequence visualization.

**Fig. 13:**
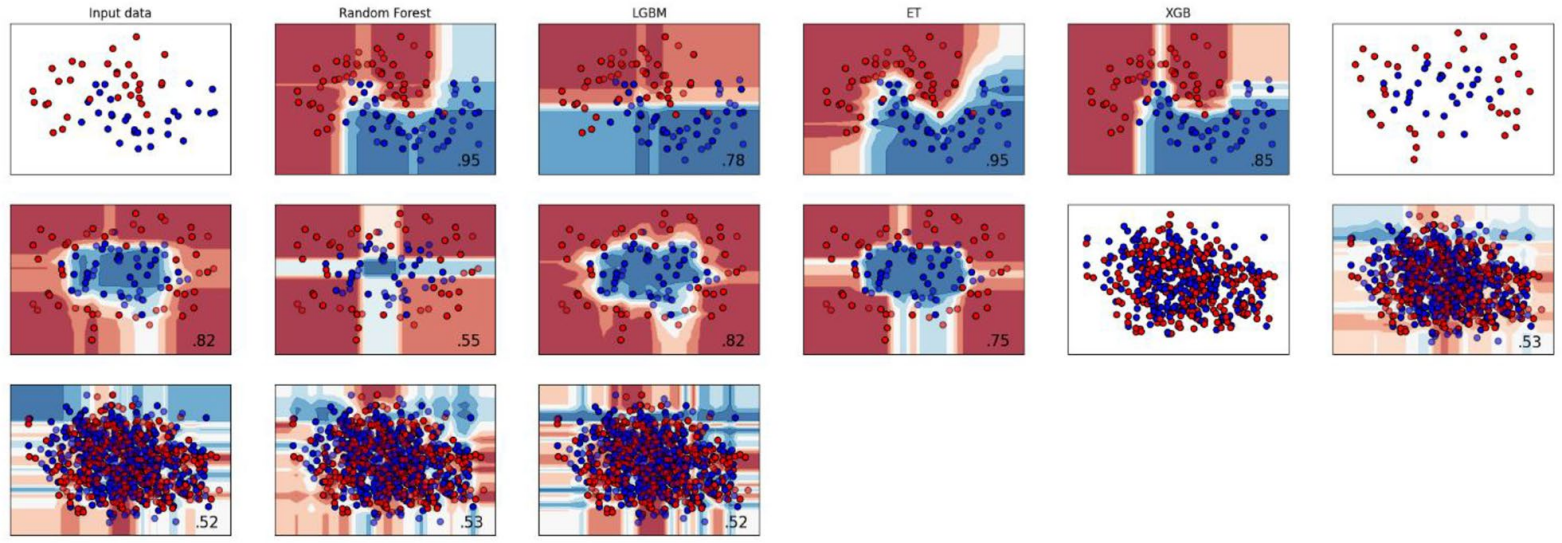
Boundary visualization for each classifier

In Fig. 13, the boundary visualization for each classifier is depicted, illustrating how they classify the positive and negative classes by creating distinct discrimination boundaries. The input data was composed of samples from both classes. After passing through heterogeneous classifiers, each classifier generated its own space for class discrimination. It is evident that the ET classifier has created a space with the fewest misclassified samples, demonstrating its effectiveness in accurately separating the classes.

**Fig. 14:**
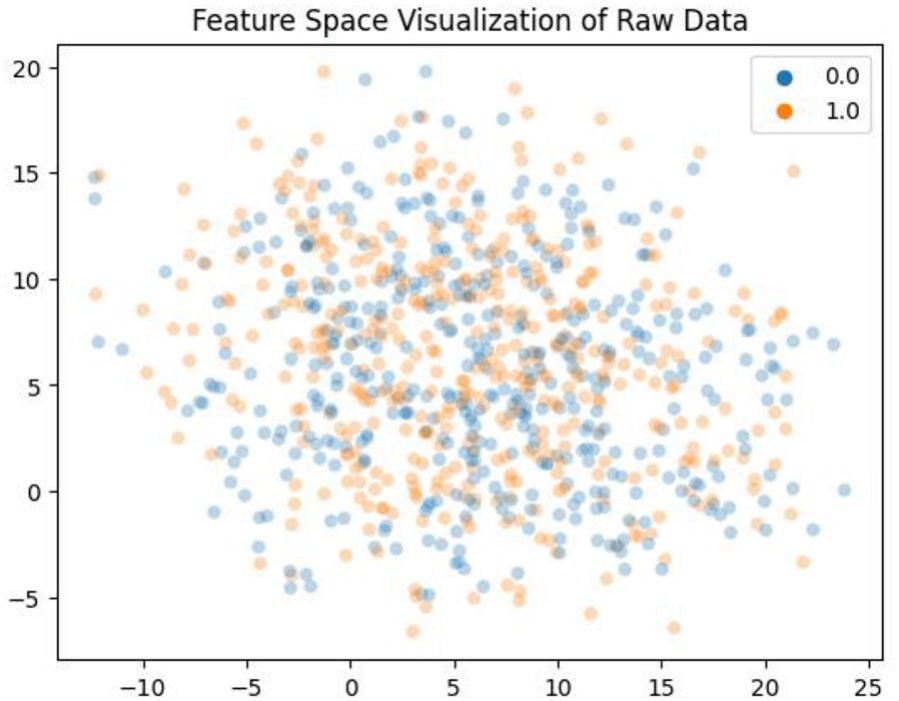
Feature space visualization

In Fig. 14, the raw feature space visualization is presented, which showcases the separability of the data. The visualization of the feature space demonstrates remarkable results, indicating that the computed features on BBB peptides hold significant discriminatory information. The data is clearly separated into two distinct clusters, representing the different classes, and can be effectively discerned by the classifiers employed in the study.

## Conclusion

This study focuses on the computational identification of BBB peptides, which may revolutionise drug delivery to the brain and open up new treatment options for disorders of the central nervous system (CNS). Peptide sequences were examined to learn more about their properties using a variety of feature computation techniques, such as PRIM, RPRIM, AAPIV, RAPPIV, and FV. Predictive models were built using ensemble-based techniques like bagging, boosting, stacking, and blending. Bagging used RF and ET classifiers, boosting used XGB and LGBM, and stacking used LR as the meta-learner in addition to ET and XGB as base learners. RF and LGBM served as the base learners in the blend, and LR served as the meta-learner. Randomized Search CV was used to optimize the hyper parameters for the XGB, LGBM, and ET. Self-consistency, independent set testing, K-fold cross-validation (5 and 10 folds), and jackknife testing, along with evaluation metrics like ACC, SPE, SEN, and MCC, were used to thoroughly evaluate the predictor’s performance. When compared to biological sequence data, the computed features show increased robustness and precision. When compared to other feature computation techniques, the proposed study accurately reflects sequence properties. As a result, the method has shown superior performance, especially in an independent set test, highlighting its improved capacity for predictive generalization. RF consistently outperformed other bagging techniques. The stacking classifier achieved high accuracy, specificity, sensitivity, MCC, and ROC score of 0.824, 0.911, 0.831, 0.663, and 0.913, respectively. Independent set testing produced the best results. Despite the fact that this study offers insightful information about BBB peptide identification, it acknowledges the drawback of not considering deep neural networks. Future studies might take into account using deep learning architectures for automated feature learning, such as FCN, 1D CNN, and RNN with GRU or LSTM.

## Data Availability

The benchmark dataset used for this study has been attached to Additional file 1. Along with the code for analysis and benchmark dataset, has been uploaded at https://github.com/Ansar390/BBB-PEP-Prediction/tree/main.

## References

[1] Sweeney MD, Zhao Z, Montagne A, Nelson AR, Zlokovic BV (2018). Blood-brain barrier: from physiology to disease and back. Physiol Rev.

[2] Abbott NJ, Patabendige AA, Dolman DE, Yusof SR, Begley DJ (2010). Structure and function of the blood–brain barrier. Neurobiol Dis.

[3] Tajes M (2014). The blood-brain barrier: structure, function and therapeutic approaches to cross it. Mol Membr Biol.

[4] Abbott NJ, Rönnbäck L, Hansson E (2006). Astrocyte–endothelial interactions at the blood–brain barrier. Nat Rev Neurosci.

[5] Friden PM (1993). Blood-brain barrier penetration and in vivo activity of an NGF conjugate. Science.

[6] Sweeney MD, Sagare AP, Zlokovic BV (2018). Blood–brain barrier breakdown in Alzheimer disease and other neurodegenerative disorders. Nat Rev Neurol.

[7] Chambers J (2012). Delivery of therapeutics to the central nervous system. Adv Drug Deliv Rev.

[8] Pardridge WM (2005). The blood-brain barrier: bottleneck in brain drug development. NeuroRx.

[9] Dai R (2021). BBPpred: sequence-based prediction of blood-brain barrier peptides with feature representation learning and logistic regression. J Chem Inf Model.

[10] Kumar V, Patiyal S, Dhall A, Sharma N, Raghava GPS (2021). B3pred: a random-forest-based method for predicting and designing blood–brain barrier penetrating peptides. Pharmaceutics.

[11] Chen X (2022). BBPpredict: a web service for identifying blood-brain barrier penetrating peptides. Front Genet.

[12] Van Dorpe S (2012). Brainpeps: the blood–brain barrier peptide database. Brain Struct Funct.

[13] Kumar V (2021). B3Pdb: an archive of blood–brain barrier-penetrating peptides. Brain Struct Funct.

[14] Awais M, Hussain W, Khan YD, Rasool N, Khan SA, Chou K-C (2019). iPhosH-PseAAC: identify phosphohistidine sites in proteins by blending statistical moments and position relative features according to the Chou’s 5-step rule and general pseudo amino acid composition. IEEE/ACM Trans Comput Biol Bioinform.

[15] Butt AH, Alkhalifah T, Alturise F, Khan YD (2023). Ensemble learning for hormone binding protein prediction: a promising approach for early diagnosis of thyroid hormone disorders in serum. Diagnostics.

[16] Ahmed S, Arif M, Kabir M, Khan K, Khan YD (2022). PredAoDP: accurate identification of antioxidant proteins by fusing different descriptors based on evolutionary information with support vector machine. Chemom Intell Lab Syst.

[17] Perveen G, Alturise F, Alkhalifah T, Daanial Khan Y (2023). Hemolytic-Pred: a machine learning-based predictor for hemolytic proteins using position and composition-based features. Digit Health.

[18] Khan YD, Alzahrani E, Alghamdi W, Ullah MZ (2020). Sequence-based identification of allergen proteins developed by integration of PseAAC and statistical moments via 5-step rule. Curr Bioinforma.

[19] Ehsan A, Mahmood MK, Khan YD, Barukab OM, Khan SA, Chou K-C (2019). iHyd-PseAAC (EPSV): identifying hydroxylation sites in proteins by extracting enhanced position and sequence variant feature via Chou’s 5-step rule and general pseudo amino acid composition. Curr Genomics.

[20] Hussain W, Rasool N, Khan YD (2020). A sequence-based predictor of Zika virus proteins developed by integration of PseAAC and statistical moments. Comb Chem High Throughput Screen.

[21] Khan YD, Khan NS, Naseer S, Butt AH (2021). iSUMOK-PseAAC: prediction of lysine sumoylation sites using statistical moments and Chou’s PseAAC. PeerJ.

[22] Butt AH, Khan YD (2020). Prediction of S-sulfenylation sites using statistical moments based features via CHOU’S 5-step rule. Int J Pept Res Ther.

[23] Butt AH, Khan YD (2019). CanLect-Pred: a cancer therapeutics tool for prediction of target cancerlectins using experiential annotated proteomic sequences. IEEE Access.

[24] AA Shah, YD Khan. SulfoTyr-PseAAC: a machine learning framework to identify sulfotyrosine sites. In 2022 International Conference on Information Science and Communications Technologies (ICISCT), IEEE, 2022, pp. 1–5.

[25] Akmal MA, Hussain W, Rasool N, Khan YD, Khan SA, Chou K-C (2020). Using Chou’s 5-steps rule to predict O-linked serine glycosylation sites by blending position relative features and statistical moment. IEEE/ACM Trans Comput Biol Bioinform.

[26] Ravichandran T, Gavahi K, Ponnambalam K, Burtea V, Mousavi SJ (2021). Ensemble-based machine learning approach for improved leak detection in water mains. J Hydroinformatics.

[27] Mehmood A (2022). Threatening URDU language detection from tweets using machine learning. Appl Sci.

[28] Deslouches B, Di YP (2017). Antimicrobial peptides with selective antitumor mechanisms: prospect for anticancer applications. Oncotarget.

[29] Farooq MS, Naseem A, Rustam F, Ashraf I (2023). Fake news detection in Urdu language using machine learning. PeerJ Comput Sci.

[30] Mosavi A, Sajedi Hosseini F, Choubin B, Goodarzi M, Dineva AA, Rafiei Sardooi E (2021). Ensemble boosting and bagging based machine learning models for groundwater potential prediction. Water Resour Manag.

[31] Liew XY, Hameed N, Clos J (2021). An investigation of XGBoost-based algorithm for breast cancer classification. Mach Learn Appl.

[32] Rahmayanti N, Pradani H, Pahlawan M, Vinarti R (2022). Comparison of machine learning algorithms to classify fetal health using cardiotocogram data. Procedia Comput Sci.

[33] Arif M (2022). StackACPred: prediction of anticancer peptides by integrating optimized multiple feature descriptors with stacked ensemble approach. Chemom Intell Lab Syst.

[34] Hansrajh A, Adeliyi TT, Wing J (2021). Detection of online fake news using blending ensemble learning. Sci Program.

[35] Ali Z, Alturise F, Alkhalifah T, Khan YD (2023). IGPred-HDnet: prediction of immunoglobulin proteins using graphical features and the hierarchal deep learning-based approach. Comput Intell Neurosci.

[36] Barukab O, Khan YD, Khan SA, Chou K-C (2022). DNAPred_Prot: identification of DNA-binding proteins using composition-and position-based features. Appl Bionics Biomech.

[37] Alzahrani E, Alghamdi W, Ullah MZ, Khan YD (2021). Identification of stress response proteins through fusion of machine learning models and statistical paradigms. Sci Rep.

[38] Almagrabi AO, Khan YD, Khan SA (2021). iPhosD-PseAAC: identification of phosphoaspartate sites in proteins using statistical moments and PseAAC. Biocell.

[39] Amanat S, Ashraf A, Hussain W, Rasool N, Khan YD (2020). Identification of lysine carboxylation sites in proteins by integrating statistical moments and position relative features via general PseAAC. Curr Bioinforma.

[40] Barukab O, Khan YD, Khan SA, Chou K-C (2019). iSulfoTyr-PseAAC: identify tyrosine sulfation sites by incorporating statistical moments via Chou’s 5-steps rule and pseudo components. Curr Genomics.

[41] Alghamdi W, Alzahrani E, Ullah MZ, Khan YD (2021). 4mC-RF: improving the prediction of 4mC sites using composition and position relative features and statistical moment. Anal Biochem.

[42] Malebary SJ, Khan YD (2021). Evaluating machine learning methodologies for identification of cancer driver genes. Sci Rep.

[43] Naseer S, Hussain W, Khan YD, Rasool N (2021). Optimization of serine phosphorylation prediction in proteins by comparing human engineered features and deep representations. Anal Biochem.

[44] Khan YD, Amin N, Hussain W, Rasool N, Khan SA, Chou K-C (2020). iProtease-PseAAC (2L): a two-layer predictor for identifying proteases and their types using Chou’s 5-step-rule and general PseAAC. Anal Biochem.

